# Clinical outcomes and coronary complication risk after transcatheter tricuspid annuloplasty with cardioband: a 12-month single-center cohort study

**DOI:** 10.1016/j.clinsp.2026.101225

**Published:** 2026-11-03

**Authors:** Hedylamar Silva de Almeida Mussons, Ángel Sánchez-Recalde, Luis Manuel Domínguez Rodríguez, José Alfredo Salinas

**Affiliations:** a Hospital zum Heiligen Geist, Krankenhaus, Germany; b Hospital Universitario Ramón y Cajal, Madrid, Spain; c Universidad de Burgos, Burgos, Spain

**Keywords:** Tricuspid valve insufficiency, Transcatheter valve repair, Annuloplasty, Right ventricular dysfunction, Heart failure

## Abstract

**Objective:**

To evaluate the clinical and echocardiographic outcomes of transcatheter tricuspid annuloplasty using the Cardioband system in a single-center cohort of high-risk patients with severe functional tricuspid regurgitation.

**Methods:**

This single-center prospective cohort study included 64 consecutive patients with severe functional tricuspid regurgitation undergoing transcatheter tricuspid annuloplasty with the Cardioband system. Clinical and echocardiographic data were collected at baseline, discharge, 6-months, and 12-months. Primary endpoints included changes in tricuspid regurgitation severity, annular dimensions, and clinical status, as well as all-cause mortality and heart failure hospitalization. Outcomes were analyzed using available-case data.

**Results:**

At 12-months, among patients with available echocardiographic data (n = 44), tricuspid regurgitation severity was reduced compared with baseline (p < 0.001); 15.9% had trace or mild TR, 45.5% had moderate TR, 27.3% had severe TR, and 11.4% had massive or torrential TR. All-cause mortality at 12-months was 6.3% (4/64 patients). According to TVARC criteria, intraprocedural success was achieved in 81.3% of patients, while a more stringent definition yielded a success rate of 65.6%.

**Conclusion:**

In this single-center cohort of high-risk patients with severe functional tricuspid regurgitation, transcatheter tricuspid annuloplasty using the Cardioband system was associated with reductions in tricuspid regurgitation severity and improvements in selected clinical parameters at 12-months. However, these findings should be interpreted with caution given the observational design, incomplete follow-up, and the absence of a control group, as well as the observed rate of procedural complications. Further studies, ideally including larger multicenter cohorts and controlled comparisons, are needed to better define the role of this therapy in the management of tricuspid regurgitation.

## Highlights

• Cardioband implantation significantly reduced tricuspid regurgitation severity at 12-months (p < 0.001).
• At 12-months, 45.5% of patients had moderate TR, while 15.9% achieved mild or trace TR.
• All-cause mortality was 6.3% and heart failure hospitalization occurred in 9.4% of patients at 12-months.
• Major adverse events occurred in 18.8% of patients, including coronary complications in 7.8%.
• Improvements in NYHA functional class and peripheral edema were observed in patients with available follow-up data.

## Introduction

Severe functional Tricuspid Regurgitation (TR) is associated with significant morbidity and mortality,^1^, ^2^, ^3^ particularly in elderly patients with multiple comorbidities. Despite its clinical impact, TR has historically been undertreated, largely due to the high surgical risk associated with conventional valve surgery in this population.^4^^,^^5^

In recent years, transcatheter therapies have emerged as potential alternatives for patients considered unsuitable for surgery. Among these, transcatheter tricuspid annuloplasty aims to reduce annular dilation and improve leaflet coaptation. Early studies have demonstrated the feasibility of these approaches; however, data on mid-term clinical and echocardiographic outcomes remain limited.^6^^,^^7^

In this context, the present study aims to evaluate the clinical and structural outcomes of transcatheter tricuspid annuloplasty using the Cardioband system in a single-center cohort of high-risk patients, with a follow-up of up to 12-months.

## Methods

### Study design and population

This was a single-center, prospective cohort study including consecutive patients with severe functional tricuspid regurgitation undergoing transcatheter tricuspid annuloplasty with the Cardioband system at Hospital Ramón y Cajal, Madrid, Spain between 2019 and 2025. This study was reported in accordance with the Strengthening the Reporting of Observational Studies in Epidemiology (STROBE) Statement.

Patients were considered eligible if they had symptomatic severe functional tricuspid regurgitation and were deemed at high or prohibitive surgical risk by the institutional heart team. All cases were discussed in a multidisciplinary setting including cardiology, cardiac surgery, and imaging specialists.

### Procedure

All procedures were performed under general anesthesia with fluoroscopic and transesophageal echocardiographic guidance. The Cardioband system (Edwards Lifesciences, Irvine, CA, USA) was implanted along the tricuspid annulus using sequential anchor deployment from the anteroseptal to the posteroseptal commissure.

After complete anchor placement, controlled cinching was performed to reduce annular dimensions and improve leaflet coaptation.

### Data collection and follow-up

Clinical and echocardiographic data were collected prospectively at baseline, discharge, 6-months, and 12-months. Follow-up was performed through scheduled outpatient visits and review of medical records (Table 1, Table 2).

**Table 1:** Baseline characteristics.

Baseline characteristics	n = 64
Patient age (years)	77.3 (7.4)
Female sex	49 (76.6%)
Height (cm)	157.5 (9.0)
Weight (kg)	65.7 (12.4)
Body mass index (BMI)	26.4 (4.3)
Atrial fibrillation	63 (98.4%)
EuroSCORE II	5.3 (3.7)
STS risk score	9.0 (5.9)
TRI risk score	4.7 (1.8)
Prior myocardial infarction	4 (6.2%)
Prior diabetes mellitus	17 (26.6%)
Prior left heart valve surgery	34 (53.1%)
Pacemaker	13 (20.3%)
Chronic obstructive pulmonary disease (COPD)	6 (9.4%)
Prior admissions for right heart failure	25 (39.1%)

**Table 2:** Baseline data.

	Summary
N	64
**Clinical status and treatment**	
NYHA functional class	
II	32 (50.0%)
III	30 (46.9%)
IV	2 (3.1%)
Ascites	18 (28.1%)
Peripheral edema	46 (71.9%)
6-minute walk distance (meters)	289.4 (109.6)
Furosemide-equivalent daily dose	66.7 (40.6)
MRA therapy	46 (71.9%)
**Laboratory**	
Albumin (g/dL)	3.9 (0.7)
Glomerular filtration rate (mL/min/1.73m^2^)	56.1 (19.7)
AST (U/L)	28.3 (11.6)
ALT (U/L)	18.2 (7.7)
GGT (U/L)	95.8 (72.2)
BNP (pre-intervention, pg/mL)	244.9 (264.9)
Hemoglobin (g/dL)	12.9 (1.7)
Total bilirubin (mg/dL)	1.3 (0.9)
**Echocardiography**	
Tricuspid regurgitation grade	
III (severe)	32 (50.0%)
IV (massive)	21 (32.8%)
V (torrential)	11 (17.2%)
Tricuspid annulus septal-lateral diameter (mm)	41.8 (6.9)
Anteroposterior annulus diameter (mm)	42.5 (6.8)
PISA effective regurgitant orifice area (mm^2^)	60.1 (31.1)
Tricuspid regurgitant volume (mL)	45.1 (19.5)
Vena contracta width (mm)	11.2 (4.1)
Tricuspid annular plane systolic excursion (mm)	18.7 (4.1)
Right ventricular ejection fraction (%)	45.3 (10.0)
Right ventricular fractional area change (%)	43.4 (8.8)
Free-wall right ventricular longitudinal strain (%)	18.6 (6.0)
Right ventricular end-diastolic volume (mL)	119.7 (43.2)
Right ventricular end-diastolic area (cm^2^)	23.8 (7.7)
Tricuspid regurgitation velocity-time integral (cm)	77.7 (17.3)
Systolic pulmonary artery pressure (mmHg)	41.5 (11.5)
Left ventricular ejection fraction (%)	57.9 (7.2)
Left ventricular end-diastolic volume (mL)	72.9 (41.3)
Left ventricular end-diastolic diameter (mm)	44.0 (8.2)
Left atrial volume (mL)	100.5 (43.1)
**Cardiac magnetic resonance imaging**	
Left ventricular ejection fraction (%, MRI)	56.7 (7.8)
Right ventricular end-diastolic volume (mL, MRI)	189.6 (61.6)
Tricuspid regurgitant volume (mL, MRI)	52.8 (25.1)
**Right heart catheterization**	
Systolic pulmonary artery pressure (mmHg)	42.9 (11.9)
Diastolic pulmonary artery pressure (mmHg)	18.7 (6.3)
Mean pulmonary artery pressure (mmHg)	26.9 (7.8)
Pulmonary capillary wedge pressure (mmHg)	17.1 (5.7)
Right ventricular systolic pressure (mmHg)	2.5 (1.2)
Cardiac index (L/min/m^2^)	2.4 (0.7)
Left ventricular end-diastolic pressure (mmHg)	17.2 (5.5)
Right ventricular end-diastolic pressure (mmHg)	13.2 (4.9)
Right atrial pressure (mmHg)	13.7 (5.4)

At 12-months, clinical follow-up data were available for 47 patients and echocardiographic data for 44 patients. The number of patients included in each analysis varied depending on data availability and is specified for each variable. No imputation for missing data was performed. Baseline right heart catheterization data were collected for descriptive characterization of the study population and were not included in outcome analyses because of the exploratory nature of the study and limited sample size (Table 3).

**Table 3:** Procedural data.

Procedural data	n = 64
Number of Cardioband anchors	15.9 (1.3)
Procedure-related hospital stay (days)	6.5 (5.1)
Procedural mortality	0 (0.0%)
In-hospital mortality	1 (1.6%)
Tricuspid regurgitation reduction (grades)	
1 grade	15 (23.4%)
2 grades	40 (62.5%)
3 grades	8 (12.5%)
4 grades	1 (1.6%)
Successful TR reduction ≥2 grades	49 (76.6%)
Procedural success^a^	52 (81.3%)
Clinical success at 30-days^b^	46/64 (71.9%)

a Procedural success (TVARC) was defined by all of the following: absence of intraprocedural mortality or stroke; successful device access, delivery, and retrieval; correct device deployment and positioning without need for unplanned devices; adequate device performance, including reduction of total tricuspid regurgitation to optimal (≤ mild [1+]) or acceptable (≤ moderate [2+]) and absence of tricuspid stenosis; no device-related obstruction of forward flow; no device-related pulmonary embolism; and no emergency surgery or reintervention within 24 hours related to the device or access.

b Clinical success at 30-days (TVARC) was defined as absence of procedural mortality or stroke; proper position of the device with adequate performance (reduction of total tricuspid regurgitation to optimal (≤ mild [1+]) or acceptable (≤ moderate [2+]) and absence of tricuspid stenosis); freedom from unplanned surgical or interventional procedures related to the device or access procedure; and absence of major device or procedure related serious adverse events.

### Endpoints

The primary endpoints of the study included echocardiographic and clinical outcomes at 12-month follow-up (Table 4).

**Table 4:** Follow-up changes.

	Baseline (n = 64)	At discharge (n = 63)	p-value (with respect to baseline)	6-months (n = 57)	p-value (with respect to baseline)	12-months (n = 45)	p-value (with respect to baseline)
**Clinical status**							
NYHA functional class							
I	0 (0.0%)					17 (36.2%)	**<0.001**
II	32 (50.0%)	‒	‒	‒	‒	18 (38.3%)	
III	30 (46.9%)					12 (25.5%)	
IV	2 (3.1%)					0 (0.0%)	
Peripheral edema	46 (71.9%)	‒	‒	‒	‒	16 (34.0%)	**<0.001**
6-minute walk test distance (m)	289.4 (109.6)	‒	‒	‒	‒	310.7 (110.7)	0.289
**Laboratory**							
Hemoglobin (g/dL)	12.9 (1.7)	11.6 (1.9)	**<0.001**	‒	‒		-
Glomerular filtration rate (mL/min/1.73m²)	56.1 (19.7)	59.2 (22.1)	0.157	‒	‒		-
BNP (pg/mL)	244.9 (264.9)	222.7 (218.8)	0.477	‒	‒	202.7 (167.2)	0.840
**Diuretic treatment**							
Furosemide-equivalent daily dose (mg) a	66.7 (40.6)	‒	‒	‒	‒	69.1 (65.4)	0.550
Mineralocorticoid receptor antagonist	46 (71.9%)	‒	‒	‒	‒	38 (59.4%)	0.077
**Echocardiography**							
Tricuspid regurgitation grade							
None/trace	0 (0.0%)	0 (0.0%)	**<0.001**	1 (2.2%)	**<0.001**	1 (2.3%)	**<0.001**
I (mild)	0 (0.0%)	29 (45.3%)		7 (15.2%)		6 (13.6%)	
II (moderate)	0 (0.0%)	23 (35.9%)		21 (45.7%)		20 (45.5%)	
III (severe)	32 (50.0%)	11 (17.2%)		11 (23.9%)		12 (27.3%)	
IV (massive)	21 (32.8%)	1 (1.6%)		5 (10.9%)		4 (9.1%)	
V (torrential)	11 (17.2%)	0 (0.0%)		1 (2.2%)		1 (2.3%)	
Tricuspid annulus septal-lateral diameter (mm)	41.8 (6.9)	30.3 (8.5)	**<0.001**	32.4 (7.0)	**<0.001**	33.3 (7.1)	<0.001
Anteroposterior annulus diameter (mm)	42.5 (6.8)	31.9 (7.4)	**<0.001**	‒	‒	‒	‒
PISA effective regurgitant orifice area (mm^2^)	60.1 (31.1)	30.7 (17.4)	**<0.001**	33.2 (21.3)	**<0.001**	36.2 (24.7)	<0.001
Vena contracta width (mm)	11.2 (4.1)	5.6 (3.2)	**<0.001**	7.4 (4.1)	**<0.001**	7.6 (3.9)	<0.001
TAPSE (mm)	18.7 (4.1)	16.2 (3.3)	**<0.001**	16.5 (4.0)	0.002	17.0 (4.8)	0.008
Right ventricular ejection fraction (%)	45.3 (10.0)	44.2 (9.3)		‒	‒	‒	
Right ventricular fractional area change (%)	43.4 (8.8)	37.3 (11.3)	0.001	37.7 (8.1)	0.001	39.3 (7.4)	0.071
Free-wall right ventricular longitudinal strain (%)	-18.6 (6.0)	-15.7 (5.3)	0.001	16.2 (6.2)	0.046	-19.3 (7.5)	0.200

Categorical variables were compared using McNemar's test for binary variables and the Wilcoxon signed-rank test for ordinal variables. Continuous variables were compared using the paired Student's *t*-test.

a 40 mg furosemide = 10 mg torasemide = 1 mg bumetanide

Echocardiographic endpoints included changes in tricuspid regurgitation severity, tricuspid annular dimensions, and right ventricular function parameters over time.

Clinical endpoints included changes in NYHA functional class, presence of peripheral edema, all-cause mortality, and hospitalization for heart failure.

Procedural outcomes included technical success and procedural success. Procedural success was defined according to the criteria of the Tricuspid Valve Academic Research Consortium (TVARC), including successful device implantation without major intraprocedural complications and achievement of adequate device performance.^8^ In addition, a more stringent definition including a reduction of ≥ 2 grades in tricuspid regurgitation was also evaluated.

Safety endpoints were defined according to TVARC criteria and included major adverse events such as mortality, life-threatening bleeding, major vascular complications, coronary complications, acute kidney injury, and device-related events (Table 5).

**Table 5:** Safety endpoints.

Safety endpoints	Early events (≤ 30 days) n = 64	Late events (31 to 365 days) n = 63^b^	Cumulative events (0 to 365 days) n = 64^b^
**Mortality and hospitalizations**			
Intraprocedural mortality	0 (0.0%)	*NA*	*NA*
In-hospital mortality	1 (1.6%)	*NA*	*NA*
All-cause mortality	1 (1.6%)	3 (4.8%)	4 (6.3%)
Cardiovascular Mortality	1 (1.6%)	2 (3.2%)	3 (4.7%)
Hospitalization for heart failure	1 (1.6%)	5 (7.9%)	6 (9.4%)
**TVARC Major Adverse Events**			
Life threatening bleeding (TVARC 5): n (%)	1 (1.6%)	0 (0.0%)	0 (0.0%)
Major vascular access complication: n (%)	3 (4.7%)	0 (0.0%)	3 (4.7%)
Major cardiac complications: n (%)	5 (7.8%)	0 (0.0%)	5 (7.8%)
Stage 2 or 3 AKI: n (%)	1 (1.6%)	0 (0.0%)	1 (1.6%)
Requiring dialysis	0 (0.0%)	0 (0.0%)	0 (0.0%)
Procedure-related myocardial infarction: n (%)	1 (1.6%)	0 (0.0%)	1 (1.6%)
Device-related obstruction of forward flow: n (%)	0 (0.0%)	0 (0.0%)	0 (0.0%)
Device-related pulmonary embolism: n (%)	1 (1.6%)	0 (0.0%)	1 (1.6%)
Hemodynamic compromise leading to major cardiac assistance	0 (0.0%)	0 (0.0%)	0 (0.0%)
Device-related dysfunction requiring for reintervention: n (%)	0 (0.0%)	2 (3.2%)	2 (3.1%)
Composite MAEs: n (%)	**11 (17.2%)**	**2 (3.2%)**	**12 (18.8%)**

Values are given as n [% with 95% Wilson confidence interval].

^a^Included in this category were patients from day-0 (day of procedure).

b Patients must have been followed for at least 31-days in this category. Composite (bold) MAE n is counted as the number of patients experiencing at least one MAE. AKI, Acute Kidney Injury; MAE, Major Adverse Event; TAVR, Tricuspid Valve Academic Research Consortium. Values are given as n [% with 95% Wilson confidence interval]. *Included in this category were patients from day 0 (day of procedure). ^⁎⁎^Patients must have been followed for at least 31 days in this category. Composite (bold) MAE n is counted as the number of patients experiencing at least one MAE. *AKI, acute kidney injury; MAE: major adverse event; TAVR: Tricuspid Valve Academic Research Consortium.* ***30-days cardiovascular mortality and life threatening bleeding (TVARC 5):*** *• Cardiovascular: gastric laceration caused by the transesophageal echocardiography probe during procedure, which led to massive upper gastrointestinal bleeding. The patient was urgently transferred to the operating room, where the bleeding could not be controlled. The patient died 24 hours after the procedure: 1* ***1-year mortality*** *• Cardiovascular* *° Heart failure: 2.* *° Gastric laceration caused by the transesophageal echocardiography probe, which led to massive upper gastrointestinal bleeding: 1* *• Non-cardiovascular* *° Traumatic brain injury.* ***Cardiac complications:*** *• Coronary perforation (required stenting): 2* *• Coronary perforation (required balloon inflation): 2* *• Posterior descending artery occlusion, conservative treatment: 1* ***Procedure-related myocardial infarction*** *• Posterior descending artery occlusion, conservative treatment: 1* ***Major access complications*** *• Femoral pseudoaneurysm requiring percutaneous intervention: 1* *• Hemoperitoneum causing hemorrhagic shock, requiring readmission within 30 days after the procedure: 1* *• Right thigh hematoma requiring blood transfusion: 1* ***Device-related pulmonary embolism*** *• Partial embolization of the device, with migration of a small metallic fragment to the pulmonary artery, which is retrieved with a snare without complications: 1* ***Device-related dysfunction requiring for reintervention at 12 months.*** *• Partial embolization of the Cardioband device during procedure. Caval valve implantation (CAVI) with TricValve device was performed 7 months later.* *• Cardioband detachment 1 month after the procedure. TricValve implantation was performed 11 months after the index procedure.* ***Device-related dysfunction requiring reintervention beyond 12 months of follow-up.*** *• Severe residual tricuspid regurgitation treated with TricValve at 13, 26 and 35 months of follow-up: 3* *• Severe residual tricuspid regurgitation treated with Evoque at 28 and 40 months of follow-up: 2*

### Statistical analysis

Continuous variables are presented as mean ± standard deviation or median (interquartile range), as appropriate. Categorical variables are presented as counts and percentages.

Comparisons between baseline and follow-up values were performed using paired Student’s *t*-test for continuous variables and the Wilcoxon signed-rank test or McNemar’s test for categorical variables, as appropriate.

Time-to-event outcomes were analyzed using the Kaplan-Meier method. Differences between crude event rates and Kaplan-Meier estimates reflect censoring and variable follow-up duration.

Given the exploratory nature of the study, the limited sample size, and the number of comparisons performed, no adjustment for multiple comparisons was applied; therefore, p-values should be interpreted as descriptive measures rather than definitive evidence of effect.

All analyses were performed using available-case data without imputation. The number of patients included in each analysis is reported for each variable and may vary across time points depending on data availability.

A two-sided p-value < 0.05 was considered statistically significant.

### Ethical considerations

The study was conducted in accordance with the principles of the Declaration of Helsinki and was approved by the Comité de Ética de la Investigación con Medicamentos (CEIm) of Hospital Universitario Ramón y Cajal, Madrid, Spain. A separate study protocol number was not available to the authors.

All patients provided written informed consent prior to inclusion in the study. This prospective observational cohort study was not registered as a clinical trial.

## Results

### Baseline characteristics

A total of 64 patients with severe functional tricuspid regurgitation were included in the study. The mean age was 77.3 ± 7.4 years, and 76.6% were female.

The study population was characterized by a high surgical risk, with a mean EuroSCORE II of 5.3 ± 3.7 and an STS score of 9.0 ± 5.9. Atrial fibrillation was present in 98.4% of patients, and more than half had a history of prior left-sided valve surgery (53.1%).

Signs and symptoms of right heart failure were common at baseline, including peripheral edema in 71.9% and ascites in 28.1% of patients. The majority of patients were in NYHA functional class II or III.

Baseline echocardiographic assessment showed severe or greater tricuspid regurgitation in all patients, with 50.0% classified as severe, 32.8% as massive, and 17.2% as torrential. Right ventricular function was moderately impaired, with a mean TAPSE of 18.7±4.1 mm (Table 1, Table 2).

### Procedural outcomes

Cardioband implantation was attempted in all patients. According to TVARC criteria, intraprocedural success was achieved in 52 patients (81.3%). When applying a more stringent definition based on a reduction of ≥ 2 grades in tricuspid regurgitation, procedural success was observed in 49 patients (76.6%) (Table 3).

Overall procedural success, defined as the combination of technical success without major intraprocedural complications and achievement of the predefined echocardiographic endpoint, was observed in 42 patients (65.6%).

The mean number of implanted anchors was 15.9±1.3.

Intraprocedural complications included coronary artery compromise in 5 patients (7.8%), which was managed during the procedure. No cases of device embolization were observed.

All patients were included in subsequent analyses regardless of procedural success.

### Echocardiographic outcomes

Echocardiographic parameters were assessed at baseline, post-procedure, 6-months, and 12-months using available-case data.

Tricuspid regurgitation severity decreased significantly following the procedure and remained reduced during follow-up (p < 0.001) (Fig. 1). At baseline, all patients had severe or greater tricuspid regurgitation. At 12-months (n = 44), 45.5% of patients had moderate regurgitation, 27.3% severe, 9.1% massive, and 2.3% torrential regurgitation.

**Fig. 1:**
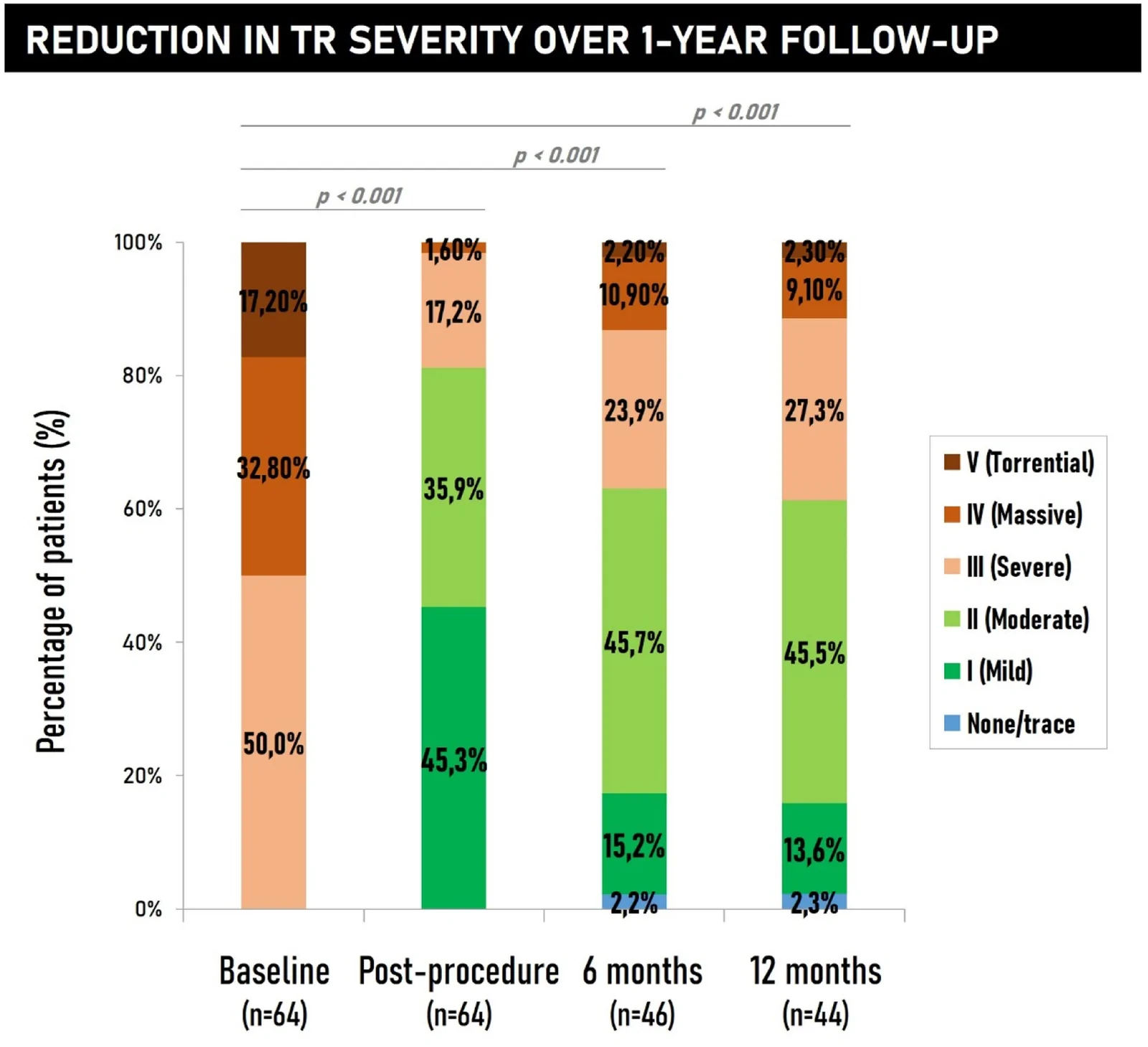
TR severity.

Tricuspid annular septal-lateral diameter decreased from 41.8±6.9 mm at baseline to 30.3±8.5 mm at discharge and remained reduced at 6-months (32.4±7.0 mm) and 12 months (33.3±7.1 mm) (p < 0.001 for all comparisons vs. baseline).

Right ventricular function parameters showed a reduction following the procedure. TAPSE decreased from 18.7±4.1 mm at baseline to 16.2±3.3 mm at discharge (p < 0.001), 16.5±4.0 mm at 6-months (p = 0.002), and 17.0±4.8 mm at 12-months (p = 0.008). Right ventricular fractional area change decreased from 43.4±8.8% at baseline to 37.3±11.3% at discharge (p = 0.001) and 37.7±8.1% at 6-months (p = 0.001), with partial recovery to 39.3±7.4% at 12-months; the difference from baseline was no longer statistically significant (p = 0.071). Estimated systolic pulmonary artery pressure showed no significant change over time (p = 0.147).

The number of patients with available echocardiographic data was 64 at baseline, 63 post-procedure, 57 at 6-months, and 44 at 12-months.

### Clinical outcomes

At 12-month follow-up, improvement in functional status was observed. The proportion of patients in NYHA functional class I increased from 0% at baseline to 36.2% at 12-months, while the proportion in NYHA class III–IV decreased from 50.0% to 25.5% (n = 64 at baseline; n = 47 at 12-months; p < 0.001) (Fig. 2 and Table 4).

**Fig. 2:**
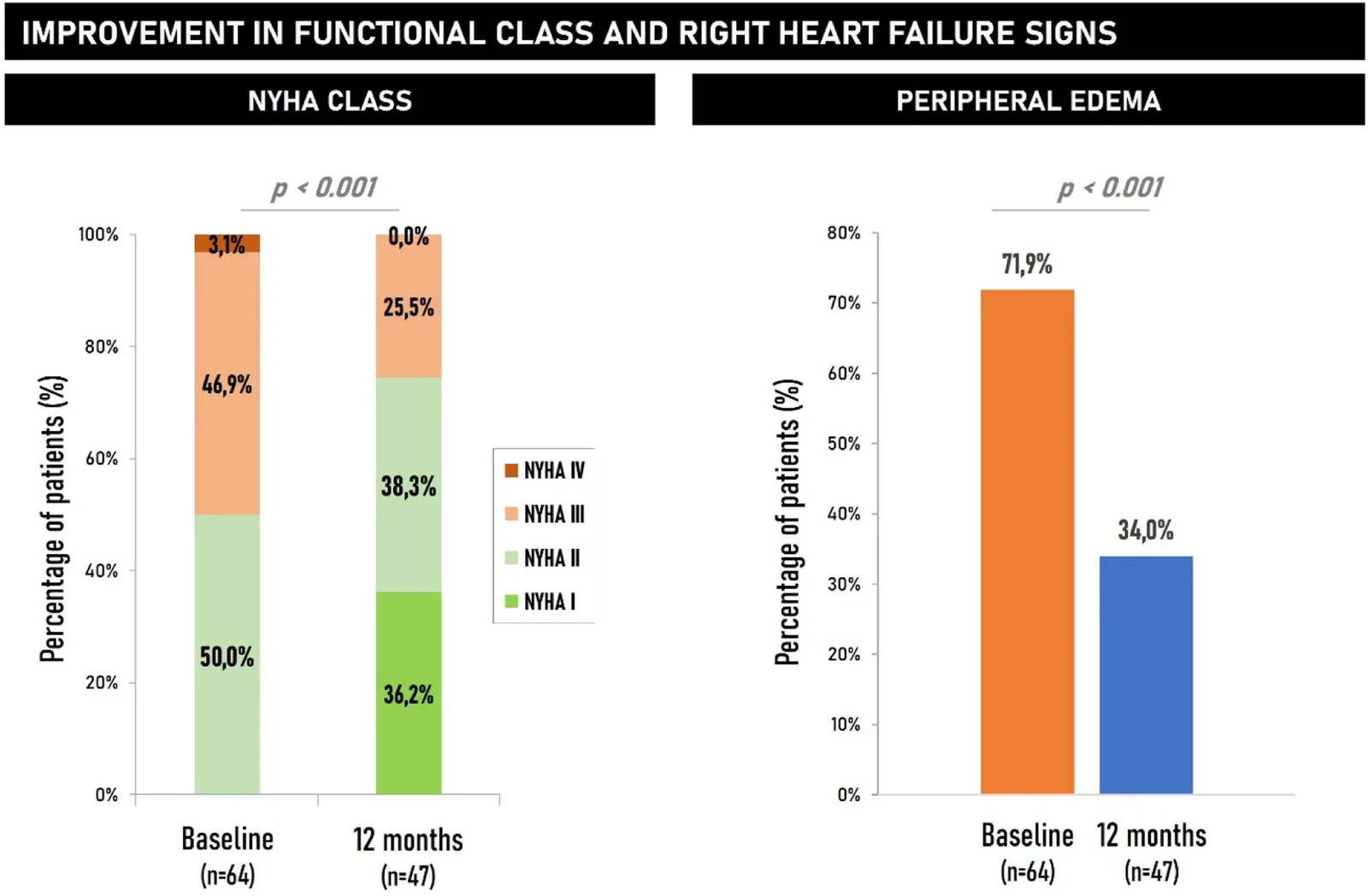
NYHA edema.

Peripheral edema was present in 71.9% of patients at baseline and decreased to 34.0% at 12-months (n = 64 at baseline; n = 47 at 12-months; p < 0.001).

All-cause mortality at 12-months was 6.3% (4/64 patients), including 3 cardiovascular deaths (4.7%). One early death was related to a procedure-associated complication (gastrointestinal bleeding secondary to transesophageal echocardiography injury). Kaplan-Meier estimates of all-cause mortality were 3.2% at 6-months and 6.9% at 12-months, with slight differences compared to crude event rates due to censoring and variable follow-up duration (Fig. 3).

**Fig. 3:**
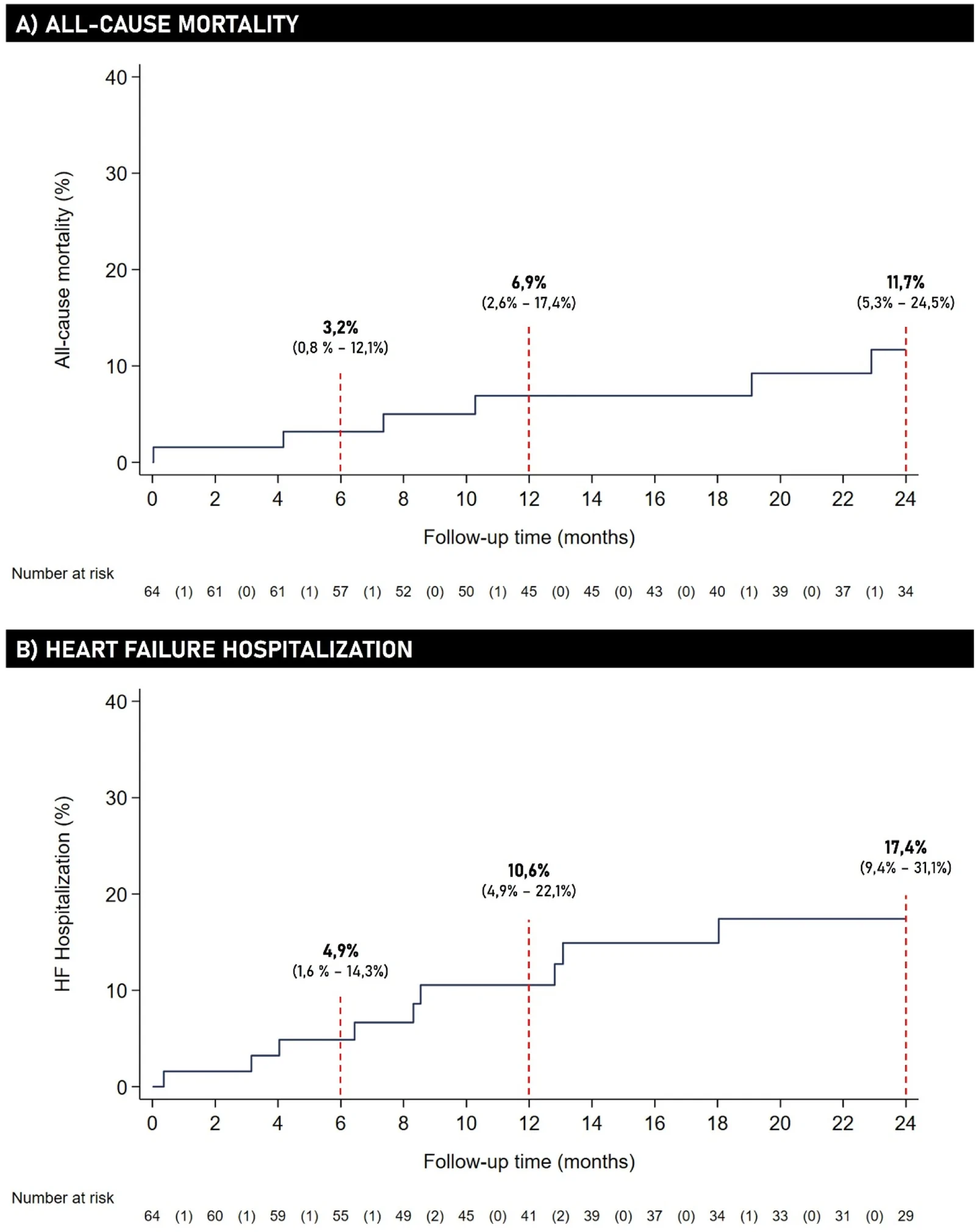
All-cause mortality and HF Hospitalization.

Heart failure hospitalization occurred in 6 patients (9.4%) at 12-months (Fig. 4).

**Fig. 4:**
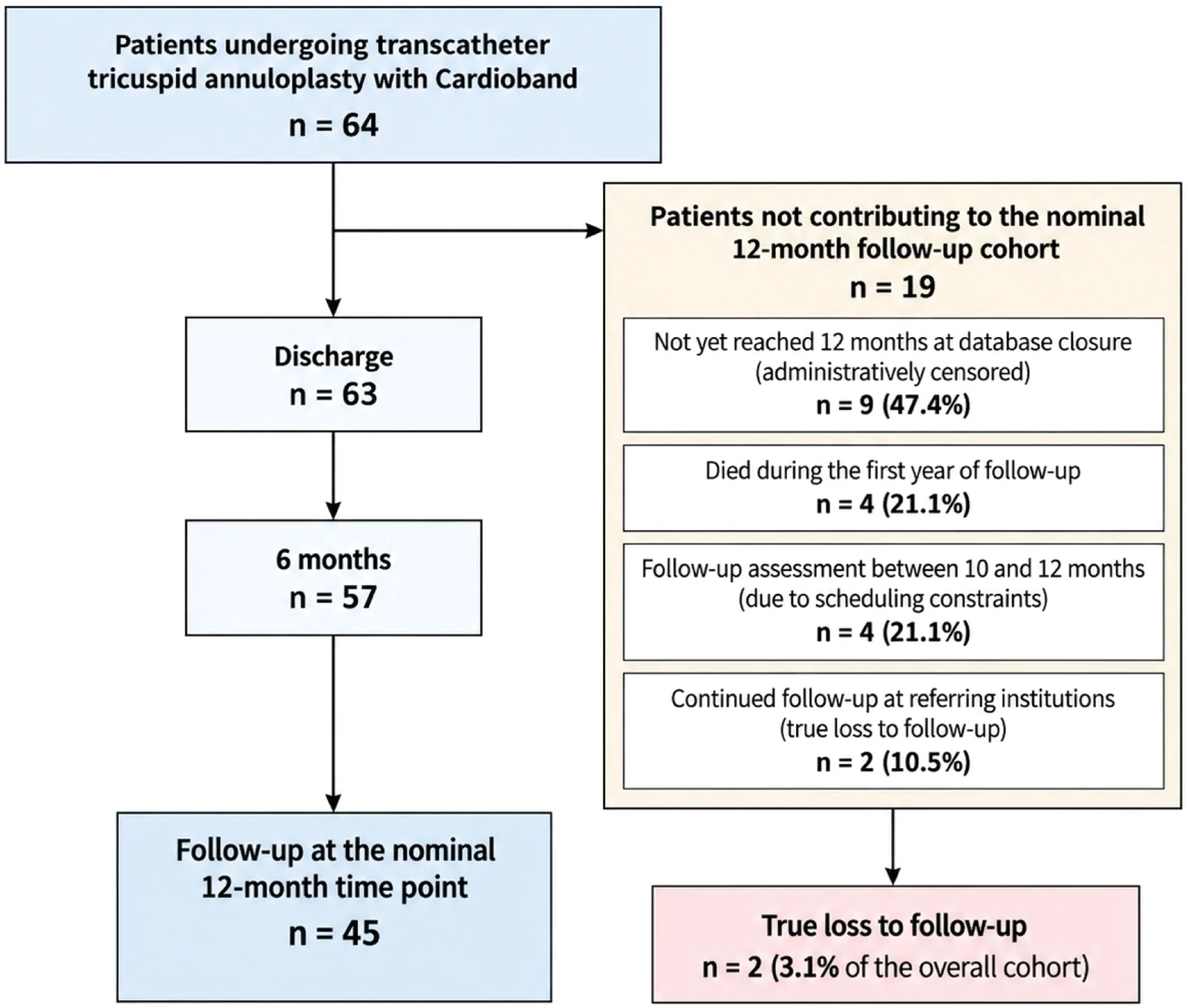
Patient follow-up flow diagram. The diagram summarizes patient during follow-up and distinguishes administrative censoring, death, assessments performed outside the nominal 12-month time point, and true loss to follow-up. Variable-specific sample sizes differed because analyses were performed using available-case data; at 12-months, clinical status data were available for 47 patients, whereas TR grade was available for 44 patients.

## Discussion

In this single-center prospective cohort of high-risk patients with severe functional tricuspid regurgitation, transcatheter tricuspid annuloplasty using the Cardioband system was associated with significant reductions in tricuspid regurgitation severity and annular dimensions, along with improvements in selected clinical parameters at 12-month follow-up.

A substantial proportion of patients achieved a reduction in tricuspid regurgitation to moderate or less following the procedure, and this reduction appeared to be maintained over time. These findings are consistent with previously published experiences with transcatheter tricuspid annuloplasty systems, including early Cardioband registries and other contemporary transcatheter therapies for tricuspid regurgitation.^6^ However, it is important to note that a considerable proportion of patients continued to exhibit moderate or greater residual regurgitation at 12-months, highlighting the complexity of this patient population and the limitations of current transcatheter approaches.

These findings should be interpreted in the context of recently published studies evaluating transcatheter therapies for tricuspid regurgitation. In the TRILUMINATE trial, transcatheter edge-to-edge repair was associated with reductions in tricuspid regurgitation severity and improvements in quality of life.^9^ Similarly, the bRIGHT study and the CLASP TR trial reported reductions in regurgitation severity and symptomatic improvement in selected high-risk populations.^10^^,^^11^

However, direct comparison between studies remains challenging due to differences in patient populations, device mechanisms, study design, and endpoint definitions. In particular, procedural success rates, safety outcomes, and residual tricuspid regurgitation vary across devices, highlighting the need for cautious interpretation and individualized patient selection.

Clinical improvement was observed in terms of NYHA functional class and reduction in peripheral edema. These findings suggest a potential symptomatic benefit associated with the intervention. Nevertheless, given the observational nature of the study and the absence of a control group, these improvements cannot be attributed solely to the device and may be influenced by other factors, including optimization of medical therapy, regression to the mean, or patient selection.

The rates of all-cause mortality (6.3% at 12-months) and heart failure hospitalization (9.4%) observed in this cohort should be interpreted with caution. Although these rates may appear relatively low in a high-risk population, the absence of a control group precludes any conclusions regarding the impact of the intervention on clinical outcomes.

Furthermore, Kaplan-Meier estimates differed slightly from crude event rates due to censoring and variable follow-up duration, which should be taken into account when interpreting these findings.

Procedural success rates varied depending on the definition applied. According to TVARC criteria, intraprocedural success was achieved in 81.3% of patients, whereas a more stringent definition incorporating a ≥ 2-grade reduction in tricuspid regurgitation resulted in a lower success rate. Importantly, patients who did not meet procedural success criteria were included in the follow-up analyses to avoid selection bias. The presence of a substantial proportion of procedural failures underscores the technical complexity of the procedure and the need for careful patient selection and procedural refinement.

The safety profile observed in this cohort warrants careful consideration. The overall rate of major adverse events at 12-months was 18.8%, including a notable incidence of coronary complications (7.8%) and one case of fatal bleeding related to transesophageal echocardiography. While some of these complications were procedure-related rather than device-specific, their occurrence highlights the invasive nature of the intervention and the potential risks associated with transcatheter tricuspid therapies. These findings emphasize the importance of procedural expertise and careful peri-procedural management. The coronary complication rate observed in this cohort may partly reflect the learning curve associated with transcatheter annuloplasty, as well as the anatomical complexity of the tricuspid annulus in patients with advanced functional TR. Increasing procedural experience and further refinement of multimodality imaging guidance may help reduce the risk of these complications.

An important observation in this study is the reduction in right ventricular function parameters, including TAPSE and right ventricular fractional area change, following the procedure. This phenomenon has been described in previous studies and may reflect an afterload mismatch following abrupt reduction in tricuspid regurgitation.^12^ Although the clinical implications of this finding remain uncertain, it underscores the need for a more comprehensive understanding of right ventricular adaptation after tricuspid interventions and warrants further investigation. Although we did not perform a formal analysis of the association between changes in right ventricular function parameters and clinical outcomes because of the limited sample size, this relationship represents an important area for future investigation in larger prospective cohorts.

While some complications were procedure-related rather than device-specific, their occurrence highlights the invasive nature of the intervention.

In conclusion, transcatheter tricuspid annuloplasty with the Cardioband system was associated with reductions in tricuspid regurgitation severity and improvements in selected clinical parameters in this high-risk population. However, these findings should be interpreted cautiously in light of the study design, incomplete follow-up, and observed safety profile. Further studies, ideally including controlled comparisons and larger multicenter cohorts, are needed to better define the role of this therapy in the management of functional tricuspid regurgitation.

### Limitations

This study has several important limitations. First, it is a single-center study with a relatively small sample size, which may limit the generalizability of the findings. Second, the absence of a control group precludes causal inference and limits the ability to attribute the observed clinical and echocardiographic improvements solely to the intervention. Factors such as optimization of medical therapy, regression to the mean, and patient selection may have influenced the results.

Third, follow-up data were incomplete, with a reduced number of patients available at 12-months. Although available-case analyses were performed, this may introduce selection bias if patients lost to follow-up differed systematically from those with complete data.

Fourth, echocardiographic assessments were performed locally without independent core laboratory adjudication, which may introduce measurement variability and potential bias in the evaluation of tricuspid regurgitation severity and right ventricular function.

Fifth, the relatively high rate of procedural and coronary complications observed in this cohort may reflect center-specific factors, operator experience, or patient selection, and should be interpreted with caution when extrapolating these results to broader populations.

Sixth, standardized quality-of-life assessments were not systematically collected, limiting the ability to fully characterize the clinical impact of the intervention.

Finally, the observational design and the absence of multivariable adjustment limit the ability to account for potential confounders; therefore, the findings should be considered hypothesis-generating.

## Clinical implications

The findings of this study provide additional real-world data on the use of transcatheter tricuspid annuloplasty in a high-risk population with severe functional tricuspid regurgitation. The observed reductions in tricuspid regurgitation severity and improvements in selected clinical parameters suggest that this approach may represent a feasible therapeutic option in carefully selected patients who are not candidates for surgery.

However, given the observational design, absence of a control group, and the variability in procedural success and safety outcomes, these results should be interpreted with caution. In particular, the persistence of moderate or greater tricuspid regurgitation in a substantial proportion of patients and the observed rate of procedural complications highlight the need for careful patient selection and procedural optimization.

These findings underscore the importance of further investigation in larger, preferably multicenter and controlled studies, to better define the role of transcatheter tricuspid annuloplasty relative to other emerging transcatheter therapies and optimized medical treatment strategies.

### Authors’ contributions

Hedylamar Silva de Almeida Mussons: Conceptualization; investigation; methodology; data curation; formal analysis; writing-original draft; writing-review & editing.

Ángel Sánchez-Recalde: Conceptualization; methodology; investigation; supervision; project administration; writing-review & editing.

Luis Manuel Domínguez Rodríguez: Methodology; formal analysis; validation; writing-review & editing.

José Alfredo Salinas: Investigation; data curation; validation; writing-review & editing.

All authors contributed to the interpretation of the results, critically reviewed the manuscript, approved the final version, and agreed to be accountable for the work.

## Funding

None.

## Data availability statement

The datasets generated and analyzed during the current study are available from the corresponding author on reasonable request.

## Conflicts of interest

The authors declare no conflicts of interest.
